# Loss of function mutation in *Ank* causes aberrant mineralization and acquisition of osteoblast-like-phenotype by the cells of the intervertebral disc

**DOI:** 10.1038/s41419-023-05893-y

**Published:** 2023-07-19

**Authors:** Takashi Ohnishi, Victoria Tran, Kimheak Sao, Pranay Ramteke, William Querido, Ruteja A. Barve, Koen van de Wetering, Makarand V. Risbud

**Affiliations:** 1 Department of Orthopaedic Surgery, Sidney Kimmel Medical College, Thomas Jefferson University, Philadelphia, PA 19107 USA; 2 Department of Orthopaedic Surgery, Faculty of Medicine and Graduate School of Medicine, Hokkaido University, Sapporo, Hokkaido 060-8638 Japan; 3 Department of Bioengineering, Temple University, Philadelphia, PA 19122 USA; 4 Department of Genetics, Genome Technology Access Centre at the McDonnell Genome Institute, Washington University, School of Medicine, St. Louis, MO 63110 USA; 5 Department of Dermatology and Cutaneous Biology, Jefferson Institute of Molecular Medicine and PXE International Center of Excellence in Research and Clinical Care, Sidney Kimmel Medical College, Thomas Jefferson University, Philadelphia, PA 19107 USA

**Keywords:** Transcriptomics, Pathogenesis

## Abstract

Pathological mineralization of intervertebral disc is debilitating and painful and linked to disc degeneration in a subset of human patients. An adenosine triphosphate efflux transporter, progressive ankylosis (ANK) is a regulator of extracellular inorganic pyrophosphate levels and plays an important role in tissue mineralization. However, the function of ANK in intervertebral disc has not been fully explored. Herein we analyzed the spinal phenotype of *Ank* mutant mice (*ank*/*ank*) with attenuated ANK function. Micro-computed tomography and histological analysis showed that loss of ANK function results in the aberrant annulus fibrosus mineralization and peripheral disc fusions with cranial to caudal progression in the spine. Vertebrae in *ank* mice exhibit elevated cortical bone mass and increased tissue non-specific alkaline phosphatase-positive endplate chondrocytes with decreased subchondral endplate porosity. The acellular dystrophic mineral inclusions in the annulus fibrosus were localized adjacent to apoptotic cells and cells that acquired osteoblast-like phenotype. Fourier transform infrared spectral imaging showed that the apatite mineral in the outer annulus fibrosus had similar chemical composition to that of vertebral bone. Transcriptomic analysis of annulus fibrosus and nucleus pulposus tissues showed changes in several biological themes with a prominent dysregulation of BMAL1/CLOCK circadian regulation. The present study provides new insights into the role of ANK in the disc tissue compartments and highlights the importance of local inorganic pyrophosphate metabolism in inhibiting the mineralization of this important connective tissue.

## Introduction

The spinal motion segment comprising the intervertebral disc and the adjacent vertebrae, absorbs the axial load and provides mobility to the spine. Calcification of the disc impairs its integrity and biomechanical function; Gruber et al. reported this incidence in 14.69% of the specimens from nonsurgical donors and tissues obtained during surgery [1]. A high prevalence of mineralization has been reported in the degenerated discs in the elderly [2–4], with males being more susceptible [5].

The balance between inorganic phosphate (Pi) and inorganic pyrophosphate (PPi) [6–8] is a critical determinant of mineralization. The progressive ankylosis gene, *Ank* is one of the important regulators of physiological mineralization modulating the Pi/PPi balance in concert with other factors [7, 9]. The occurrence of ectopic mineralization suggests disruption of this regulatory mechanism, which may occur in the disc with aging [8, 9]. Interestingly, increased levels of ANK protein were reported in degenerated human NP [10], suggesting a possible compensation to adjust the local Pi/PPi balance. Loss of ANK function also results in ectopic mineralization of the articular cartilage and other joint tissues, resulting in progressive loss of joint mobility in *Ank* loss of function, *ank*, mice [11]. In addition, ANK is known to elevate extracellular PPi which inhibits hydroxyapatite deposition by preventing the growth of nascent Ca_3_(PO_4_)_2_ crystals [12]. Similar to its role in cartilage, ANK is regarded as an important regulator of calcification and development of bone tissue. While *Ank* knockdown decreased the expression of osteoblast marker genes *Alpl*, *Ibsp*, and *Sp7*, its overexpression increased these markers in preosteoblastic MC3T3-E1 cells [13, 14]. In *ank* mice, parameters of trabecular bone quality in the femur and tibia are affected [14] with a decrease in bone volume and trabecular thickness with increased trabecular separation, without an effect on cortical thickness [14]. These studies indicate that on the one hand, ANK prevents mineralization in the cartilage while on the other hand acts as a pro-mineralization factor in the bone.

Sampson and Davis reported the disc mineralization in *ank* mice before ANK function was discovered at the molecular level without elucidating the cellular and molecular mechanisms of aberrant mineralization [15, 16]. We, therefore, investigated the spinal phenotype of *ank* mice to delineate cellular and molecular mechanisms that underscore the phenotype. Our findings provide new insights into the importance of ANK in the maintenance of spine health and its role in preventing pathological mineralization of the disc.

## Results

### ank mice show intervertebral disc fusion and altered vertebral bone parameters

**Fig. 1:**
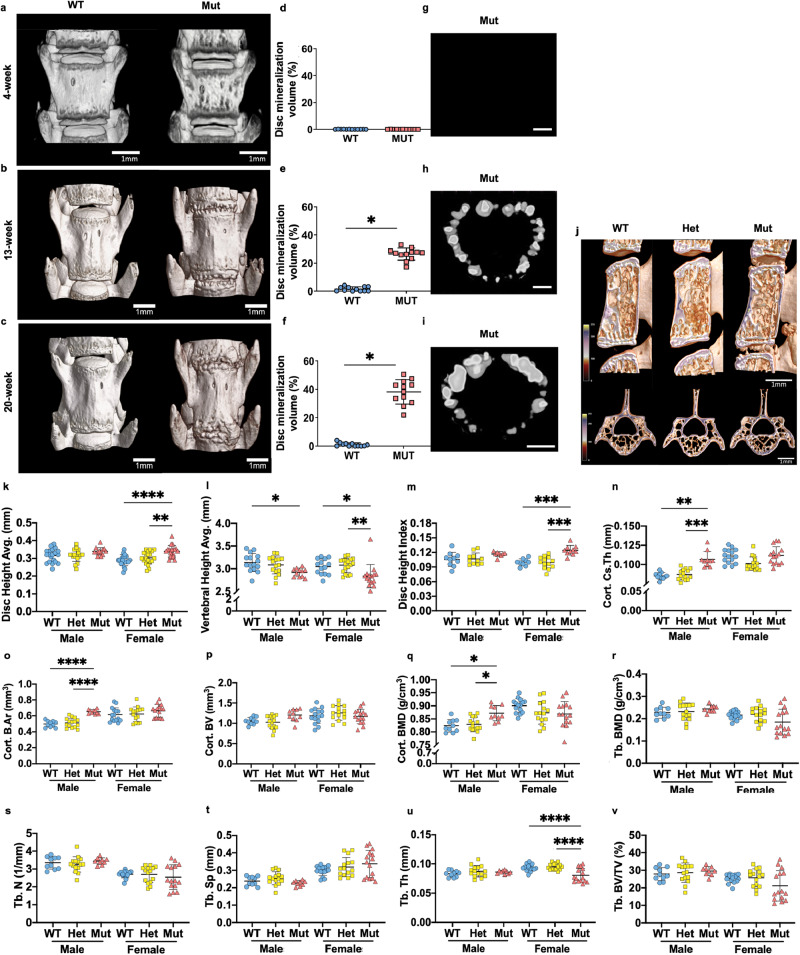
The *ank* mutants presented greater disc heights and more bone mass in the cortical bone. **a**–**c** Representative micro-CT images of 4-week-old (**a**), 13-week-old (**b**), and 20-week-old (**c**) WT and *ank* mutant (Mut) lumbar spines. The center vertebra corresponds to L4. Scale bars, 1 mm. **d–i** Disc mineralization volume quantifications and images in the lumbar discs of WT and Mut mice of **d** 4-week-old, **e** 13-week-old, and **f** 20-week-old. Transaxially cut surface rendering images of the L6/S disc of **g** 4-week-old, **h** 13-week-old, and **i** 20-week-old Mut mice. Scale bars, 250 μm. **j** Sagittally and transaxially cut surface rendering images with a heatmap of bone mass density. Scale bars, 1 mm. **k** Disc heights of L4/5, L5/6, and L6/S1. **l** Vertebral heights of L4, L5, and L6 vertebrae. **m** Disc height indices of L4/5 and L5/6 discs. **n** Cortical cross-sectional thickness (Cort. Cs. Th) (mm); **o** Cortical bone area (Cort. B. Ar) (mm^2^); **p** Cortical bone volume (Cort. BV) (mm^3^); **q** Cortical bone mass density (Cort. BMD) (g/cm^3^). **r** Trabecular bone mass density (Tb. BMD) (g/cm^3^). **s** Trabecular number (Tb. N) (1/mm); **t** Trabecular spacing (Tb. Sp) (mm); **u** Trabecular thickness (Tb. Th) (mm); **v** Trabecular bone volume/tissue volume (Tb. BV/TV) (%); L4-6 vertebrae were analyzed for bone parameters. WT: 7–10 mice (4–5 male; 3–5 female); Het: ten mice (five male; five female); Mut: eight mice (three male; five female). Quantitative measurements represent mean ± SD. Significance was tested using ANOVA or Kruskal–Wallis test followed by Dunn’s multiple comparison test. *p < 0.05, **p < 0.01, ***p < 0.001, ****p < 0.0001.

Micro-CT (μCT) imaging was performed to visualize the spines of 4-, 13-, and 20-week-old *Ank*^*ank/ank*^ (*ank*), and *Ank*^*+/+*^ (wildtype) mice. It was evident that *ank* mice suffered from aberrant mineralization in the disc at 13 weeks, with fusion and peripheral bridging, features not seen in 4-week-old animals. The phenotype was progressive with age and mineralization appeared to be restricted to the AF (Fig. 1a–i). Importantly, the cervical, thoracic, and lumbar spine was prominently affected without a noticeable effect on the caudal region at 13 weeks of age, with the more caudal disc being affected at 20 weeks, suggesting cranial to the caudal progression of the pathological disc mineralization (Supplemental Fig. S1a, b). The mineral volume within the discs was quantified at 4-, 13-, and 20-weeks, showing significant mineralization only in the *ank* mice (Fig. 1d–f). Analyses of 13-week-old *Ank*^*ank/ank*^*, Ank*^*ank/+*^(Het), and WT mice showed that female *ank* mice had an increase in disc height, smaller vertebral heights, and greater disc height indices (DHI) (Fig. 1j–m). Whereas, cortical thickness, bone area, bone volume, and bone mineral density (BMD) were all significantly greater in male mutants suggesting an increased cortical bone mass (Fig. 1n–q), similar to an increase in BMD reported in an osteoblast-specific *Enpp1*-KO female mice [17]. Interestingly, unlike findings in long bones, trabecular number, trabecular separation, bone volume/tissue volume (BV/TV), and BMD were similar between genotypes, except for significantly smaller trabecular thickness in female mutant mice (Fig. 1r–v) [14].

### Dystrophic mineral deposits in the AF compartment of ank mice show regional differences in composition

**Fig. 2:**
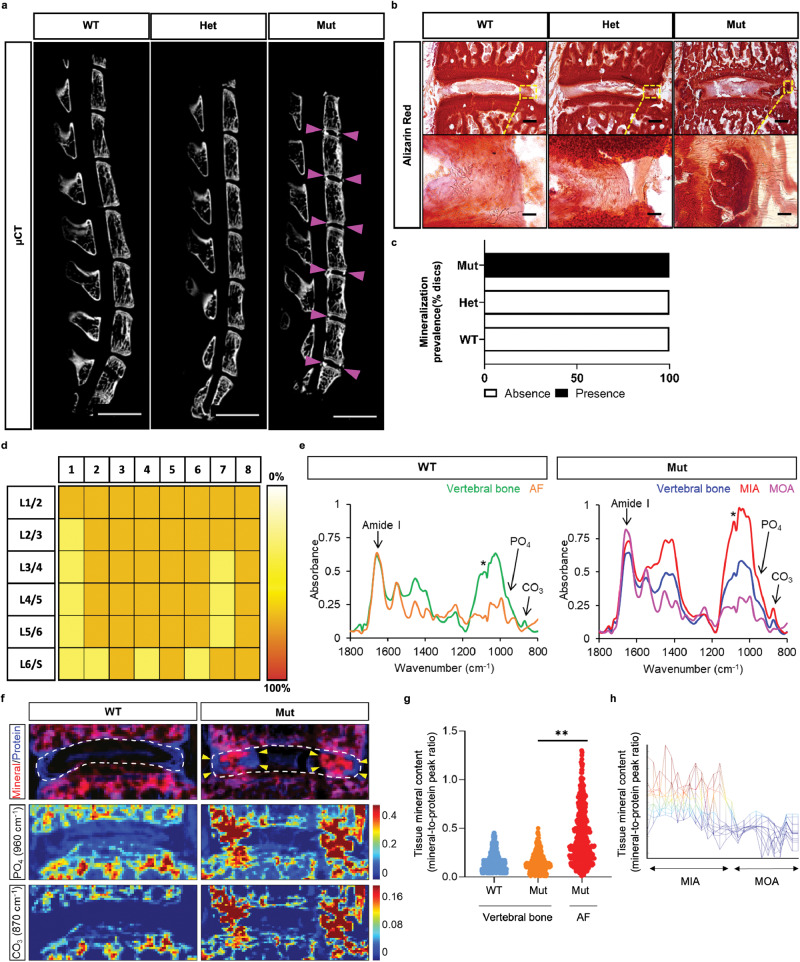
*ank* mutants manifest compositionally heterogenous calcified mineralization in the AF. **a** Micro**-**CT showed higher prevalence and widespread distribution of disc calcification in the lumbar spine of 13-week-old mutants. Arrowheads indicate the ectopic mineralization of the AF. Scale bar, 4 mm. **b** Alizarin Red staining showed staining of the mutant AF. Scale bars, 200 μm for low magnification and 20 μm for high magnification images. **c** Prevalence of calcified mineralization in the disc. WT and Het, 0/10 mice; Mut, 8/8 mice. **d** Color scale represents small, medium, and large-size ectopic mineral deposits in the lumbar discs of eight mutants. **e** FTIR spectra show absorbance bands from collagen (amide I at 1660 cm^−1^) and apatite (phosphate at 960 cm^−1^ and carbonate at 870 cm^−1^) in the vertebral bones. In Mut animals, intense apatite bands can also be seen in the intermediate layer of the AF (MIA), but not in the outer layer of the AF (MOA). *Artifact from spectral subtraction of cryotape signals. **f** FTIR spectral imaging shows the overlaid distribution of mineral and protein, highlighting the clear mineralization of the MIA of Mut animals. Dashed white lines demark the intervertebral disc, yellow arrowheads point to the mineralized AF in Mut animals. The distribution of phosphate and carbonate appears more intense in the AF mineralization. The color scale shows spectral intensity at specific absorbance bands, reflecting the presence of corresponding components. **g** Assessment of the tissue mineral content by quantification of the mineral-to-protein (960/1660) peak intensity ratios, from inverted second derivatives of the spectra. Whereas the mineral content was similar in the vertebral bones of WT and Mut animals, the MIA had significantly greater mineral content (**p < 0.01). **h** Mineral content across the MIA-MOA interface illustrates the prevalence of mineralization in the MIA and its absence in the MOA.

Disc mineralization phenotype in ank mice had 100% penetrance regardless of their sex at 13 weeks and beyond. Similarly, all the lumbar discs (L1/2 - L6/S1) in 13-week-old ank mice but none in WT and Het mice evidenced mineralization (Fig. 2a–d). Mineralized, frozen sections of spinal motion segments from 13-week-old mice stained with Alizarin Red showed that mineralization affected only the AF but not the NP compartment in ank mice (Fig. 2b). This was noteworthy since analyses of the ScRNA-Seq data from the rat (GSE211407) [18] and bovine (GSE179714) [19] discs showed expression of Ank by NP and AF cells (Supplemental Fig. S2a–d). Similarly, ScRNA-Seq data from healthy human NP tissue (GSE205535) showed prominent ANKH expression by ACAN-expressing NP cell clusters [20] (Supplemental Fig. S2e, f) supporting our previous findings in the mouse and human [10].

Subsequently, Fourier transform infrared (FTIR) spectral imaging was performed to compare the chemical composition of intradiscal minerals with that of vertebral bone [21]. FTIR spectra (Fig. 2e) of the vertebral bone of WT and *ank* (Mut) animals showed typical absorbances from collagen (Amide I at 1665 cm^−1^) and carbonated apatite mineral (PO_4_^3−^ at 960 cm^−1^ and CO_3_^−^ at 870 cm^−1^). In WT animals, as expected, no absorbance bands from phosphate or carbonate were seen. In contrast, *ank* animals showed intense mineralization in the intermediate layer of the AF (MIA), which was not obvious in the outer layer of the AF (MOA). FTIR imaging (Fig. 2f) further demonstrated these findings, illustrating the carbonate-containing basic calcium phosphate apatite deposition in the MIA of Mut animals. The overlay of the mineral and protein FTIR images did not detect a prominent amount of apatite in the MOA compared to that in the MIA. Moreover, FTIR imaging of phosphate and carbonate highlighted the intense apatite deposition in the MIA of *ank* animals. In fact, spectral quantification of the mineral-to-protein peak ratio (Fig. 2g) showed a significantly greater tissue apatite content in the MIA of *ank* animals than in the vertebral bone of either WT and *ank* animals, suggesting that the composition of the MIA was discrete from that of native bone matrix. In addition, spectral quantification of mineral content across the MIA-MOA interface (Fig. 2h) demonstrated the different mineralization prevalence in these regions.

### Loss of Ank function causes structural disruption of disc compartments, disorganization of the AF matrix, and promotes degeneration

**Fig. 3:**
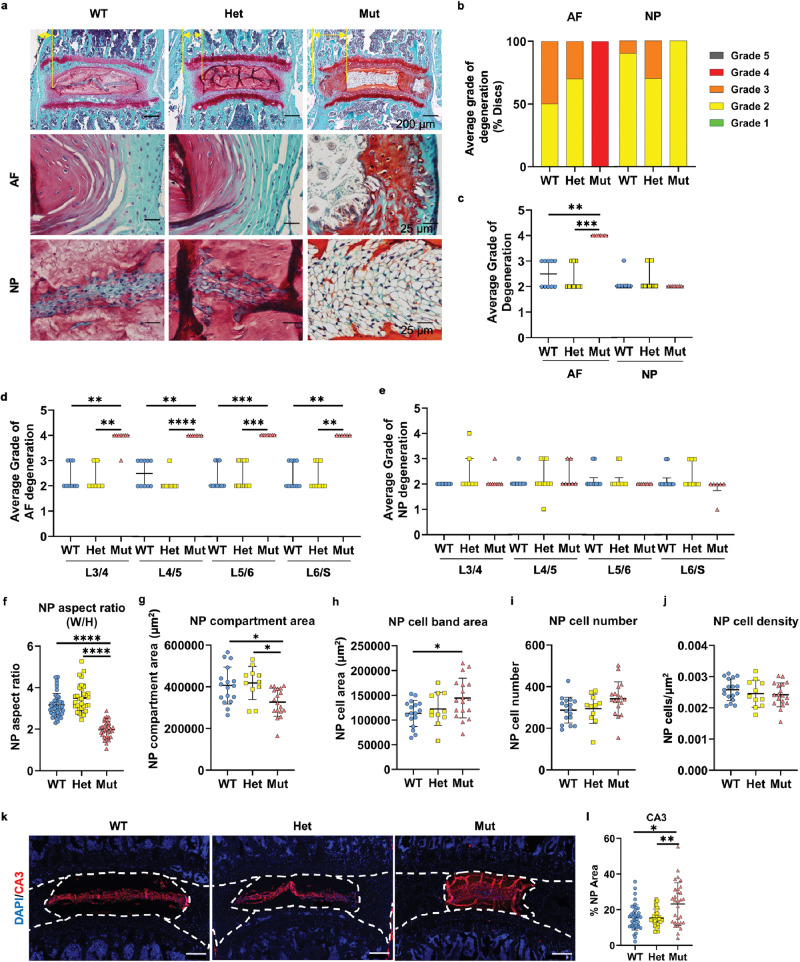
Disorganized and enlarged AF, and smaller compartment but maintained phenotype of the NP in *ank* mutants. **a** Histology of 13-week-old mice showing differences in tissue structure and cell morphology in the AF and NP. **b**–**e** Modified Thompson Grading distribution and averages showed small degenerative changes in the NP but high degenerative grades in the AF regardless of the levels. WT: 11 mice (six male; five female); Het: nine mice (four male; five female); Mut: nine mice (three male; six female), 3–4 discs/mouse. **f**–**j** Analyses of NP morphology, NP area, cell band area, cell number, and cell density. **k**, **l**. Carbonic anhydrase 3 (CA3) represents the NP phenotype. WT: 11 mice (six male; five female); Het: nine mice (four male; five female); Mut: nine mice (three male; five female), 3–4 discs/mouse. Dotted lines demarcate different tissue compartments within the disc. Quantitative measurements represent mean ± SD. Significance was tested using ANOVA or Kruskal–Wallis test followed by Dunnett’s T3 multiple comparisons test or Dunn’s multiple comparison test. *p < 0.05, **p < 0.01, ***p < 0.001, ****p < 0.0001.

Since the mineralization phenotype in intervertebral discs of *ank* mice showed 100% penetrance and was independent of the sex, the male and female data were analyzed together. Histological findings from 13-week-old mice showed significant changes to the AF matrix structure and organization in *ank* mice. The AF lamellae showed signs of buckling, cellular disorganization in the outer and mid-AF regions, and overall AF hyperplasia (Fig. 3a–d). NP cells appeared turgor and full of vacuoles as opposed to a compact morphology in WT and Het animals, suggesting diminished loading of the disc (Fig. 3a–c, e). There was a concomitant decrease in the aspect ratio and the area of the NP in *ank* mutants (Fig. 3f, g). While the NP cell band had a larger area, the cell number or cell density was similar among genotypes (Fig. 3h–j). Despite these morphological changes suggesting pathological alterations, levels of carbonic anhydrase 3 (CA3), an NP phenotypic marker, were maintained (Fig. 3k, l).

### *ank* mice show alteration in endplate morphology

**Fig. 4:**
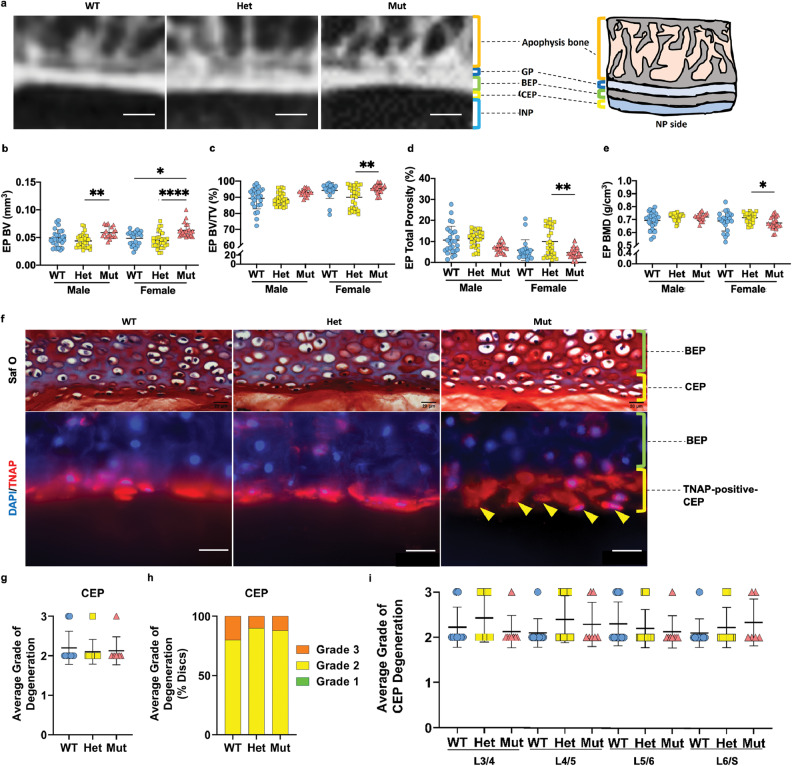
More bone mass in the BEP and thicker but organized CEP in *ank* mutants. **a** Representative micro-CT images and cartoon illustration of the L4/5 disc upper EP. Scale bar, 200 μm. GP growth plate. **b** EP bone volume (EP BV) (mm^3^). **c** EP bone volume/tissue volume (EP BV/TV) (%). **d** EP total porosity (%). **e** EP bone mass density (EP BMD) (g/cm^3^). L4, 5, 6 vertebrae were analyzed for bone parameters. WT: 7–10 mice (4–5 male; 3–5 female); Het: ten mice (five male; five female); Mut: eight mice (three male; five female). Quantitative measurements represent mean ± SD. Significance was tested using ANOVA or Kruskal–Wallis test followed by Dunn’s multiple comparison test. *p < 0.05, **p < 0.01, ****p < 0.0001. **f** Representative images of Safranin O/Fast Green staining and TNAP staining of the EP. Arrowheads indicate an additional layer of cells. Scale bar, 200 μm. **g**–**i** Histological grading assessment as described by Tessier et al. WT: 11 mice (six male; five female); Het: nine mice (four male; five female); Mut: nine mice (three male; five female), 3–4 discs/mouse. Significance was tested using ANOVA or Kruskal–Wallis test followed by Dunnett’s T3 multiple comparisons test or Dunn’s multiple comparison test. *p < 0.05.

The endplate (EP) consists of a subchondral bony EP (BEP) (calcified cartilage before skeletal maturity) and an uncalcified thin layer of cartilaginous EP (CEP) which interfaces with the NP and AF (Fig. 4a) and is critical for nutrient diffusion [22]. μCT analyses showed changes in the BEP parameters in mutants (Fig. 4b–e). Notably, histologically there were no defects in the EP of *ank* mice and the gross morphology of the CEP was similar between genotypes. However, the formation of secondary ossification centers in the BEP was delayed in the mutants (Fig. 3a) and showed increased EP cellularity (Fig. 4f). EP scoring showed similar distribution and average scores of degeneration (Fig. 4g–i). Supporting the observation of Safranin O/Fast Green staining, there was an increase in the number of TNAP-positive CEP cells which resulted in the thickening of the CEP in mutants (Fig. 4f). These results suggested that loss of *Ank* function affects the EP structure.

### *ank* mice show altered disc matrix composition

**Fig. 5:**
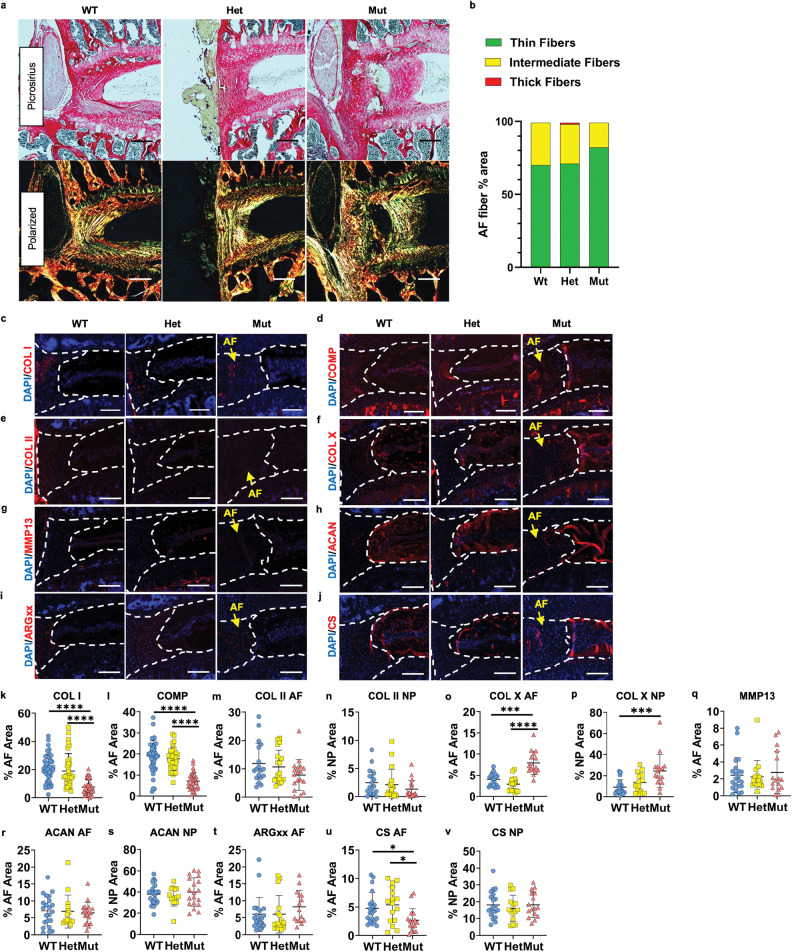
Alteration in the collagen composition and turnover as well as changes in the CS amount in the AF matrix of *ank* mutants. **a** Picrosirius Red staining and polarized microscopy of discs. Scale bar, 200 μm. **b** Analysis of the percentage of thin (green), intermediate (yellow), and thick fibers (red). The chi-square test was used for statistical analysis. WT: 11 mice (six male; five female); Het: nine mice (four male; five female); Mut: nine mice (three male; five female), 3–4 discs/mouse. **c**–**v** Quantitative immunohistochemical staining of matrix components and catabolism markers: **c**, **k** COL I; **d**, **l** COMP; WT: 5–9 mice (3–5 male; 2–5 female); Het: 4–8 mice (2–4 male; 2–5 female); Mut: nine mice (three male; five female), 2–4 discs/mouse. **e**, **m**, **n** COL II; **f**, **o**, **p** COL X; **g**, **q** MMP13; **h**, **r**, **s** ACAN; **i**, **t** ARGxx; **j**, **u**, **v** CS; WT: 5–9 mice (six male; five female); Het: nine mice (four male; five female); Mut: 4–7 mice (2–3 male; 1–5 female), 2–4 discs/mouse. Dotted lines demarcate different tissue compartments within the disc. Scale bar, 200 μm. Quantitative measurements represent mean ± SD. Significance was tested using ANOVA or Kruskal–Wallis test followed by Dunnett’s T3 multiple comparisons test or Dunn’s multiple comparison test. *p < 0.05, ***p < 0.001, ****p < 0.0001.

There was a trend towards a higher ratio of green fibers in the AF of mutants in Picrosirius Red staining, suggesting collagen fibers were less cross-linked and showed a higher turnover rate (Fig. 5a, b). Immunohistochemical analyses revealed that the abundance of collagen I (COL I) and cartilage oligomeric matrix protein (COMP) was significantly lower in the AF compartment of mutants (Fig. 5c, d, k, l). Collagen II (COL II) staining, however, did not differ among genotypes in both AF and NP (Fig. 5e, m, n). Our results showed a higher abundance of collagen X (COL X) in mutants, without simultaneously increased MMP13 staining (Fig. 5f, g, o–q). Therefore, an increase in COL X could be a compensatory response to modulate ectopic mineralization [23], and not a sign of hypertrophic chondrocytes [24]. Although we observed similar aggrecan (ACAN) and ARGxx staining among genotypes (Fig. 5h, i, r–t), chondroitin sulfate (CS)-positive areas were smaller in mutants (Fig. 5j, u, v), indicating diminished CS substitution in CS-enriched proteoglycans, including ACAN. Collectively these results suggest that the composition of the disc matrix of *ank* mice is altered, resulting in compromised tissue function.

### AF cells of *ank* mice show increased apoptosis and acquire an osteoblast-like phenotype

**Fig. 6:**
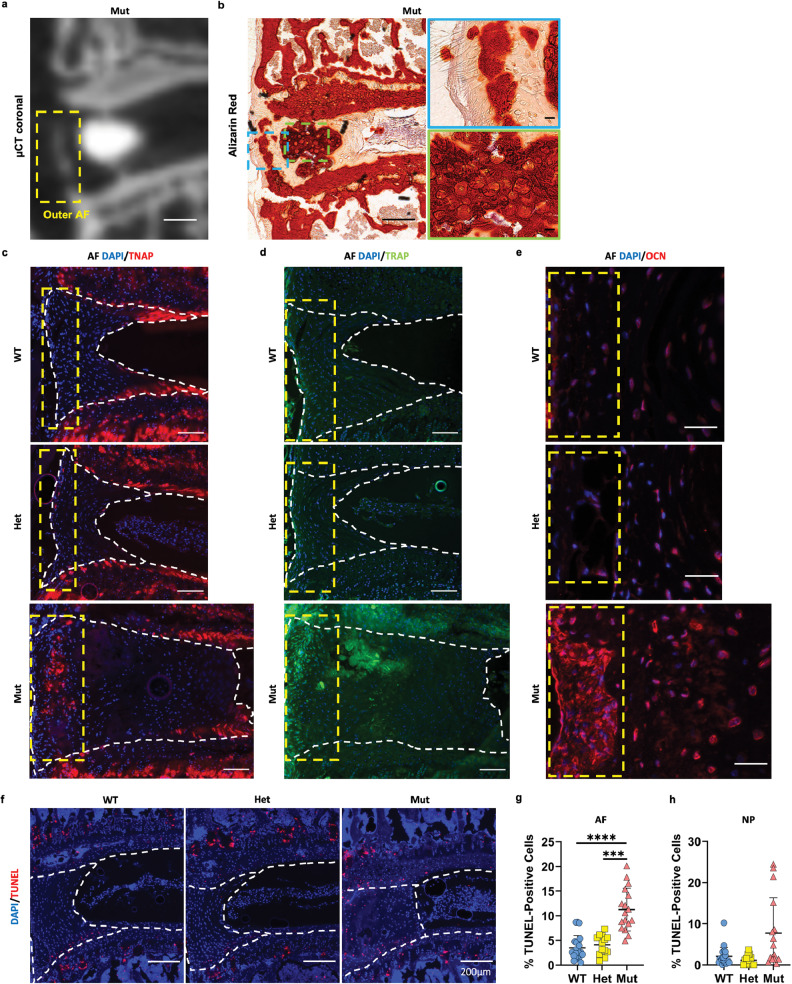
Acquisition of osteoblast-like-phenotype in the cells of the outer AF with cell death in the intermediate AF and NP in *ank* mutants. **a**–**d** Qualitative analyses of the calcified mineralization in the same AF from the same mutant: **a** μCT; **b** Alizarin Red staining; **c** TNAP staining; **d** TRAP staining. Scale bars, 200 μm for low magnification and 20 μm for high magnification images. **e** Immunohistochemical analysis of osteocalcin (OCN) in the mutant AF. Scale bars, 25 μm. Yellow dotted lines demarcate the outer AF (**a**, **c**–**e**). **f**–**h** TUNEL assay showing greater numbers of cells showing positivity in the AF and NP of mutants. Scale bars, 200 μm. WT: nine mice (five male; four female); Het: seven mice (two male; five female); Mut: 7–8 mice (2–3 male; five female), 1–4 discs/mouse. White dotted lines demarcate different tissue compartments within the disc. Quantitative measurements represent mean ± SD. Significance was tested using Kruskal–Wallis test followed by Dunn’s multiple comparison test. ***p < 0.001, ****p < 0.0001.

Based on FTIR spectroscopic and immunohistology, we hypothesized that the mineralization in the MIA and MOA may represent discrete mineralization phases. The μCT scans showed that MIA exhibited higher radio-opacity compared to that of the MOA which was comparable to that of the bone (Fig. 6a). Alizarin Red staining on the corresponding discs revealed that staining of the MIA was heterogenous and disorganized, while that of the MOA was along the lamellae (Fig. 6b). In the same discs TNAP and TRAP-positive cells were identified in the MOA and almost no labeling was seen in the MIA region (Fig. 6c, d). In addition, the MOA region showed robust cellular osteocalcin (OCN) staining (Fig. 6e) without any differences in Ki67 staining among genotypes (Supplemental Fig. S3a, b). We further analyzed mutant discs for cathepsin K (CTSK), CD68, and CD14, markers of osteoclasts, macrophages/osteoclasts, and macrophages, respectively [25–28]. These molecules were not expressed by the cells in the MOA region (Supplemental Fig. S3c–e), suggesting that the cells are not likely to be osteoclasts despite TRAP positivity. We further investigated whether endochondral ossification occurred in the region of the MOA but did not observe substantial CD31 or Indian hedgehog (IHH) staining in the region of the MOA (Supplemental Fig. S3f, g). This indicates that a population of resident AF cells acquired osteoblast-like-phenotype in the absence of overt endochondral ossification and laid down bone-like-matrix in the region of the MOA. Finally, TUNEL-staining demonstrated increased apoptosis in the AF of *ank* mutants, and apoptotic cells primarily localized to the periphery of the acellular mineral inclusion in the intermediate-annulus (Fig. 6f, g). There was also a slight increase in TUNEL-positive cells in the NP compartment of the *ank* mutant (Fig. 6f, h).

### Global transcriptomic analyses show significant alterations in AF and NP of ank mutants with a pronounced perturbation in BMAL1/CLOCK circadian regulation

**Fig. 7:**
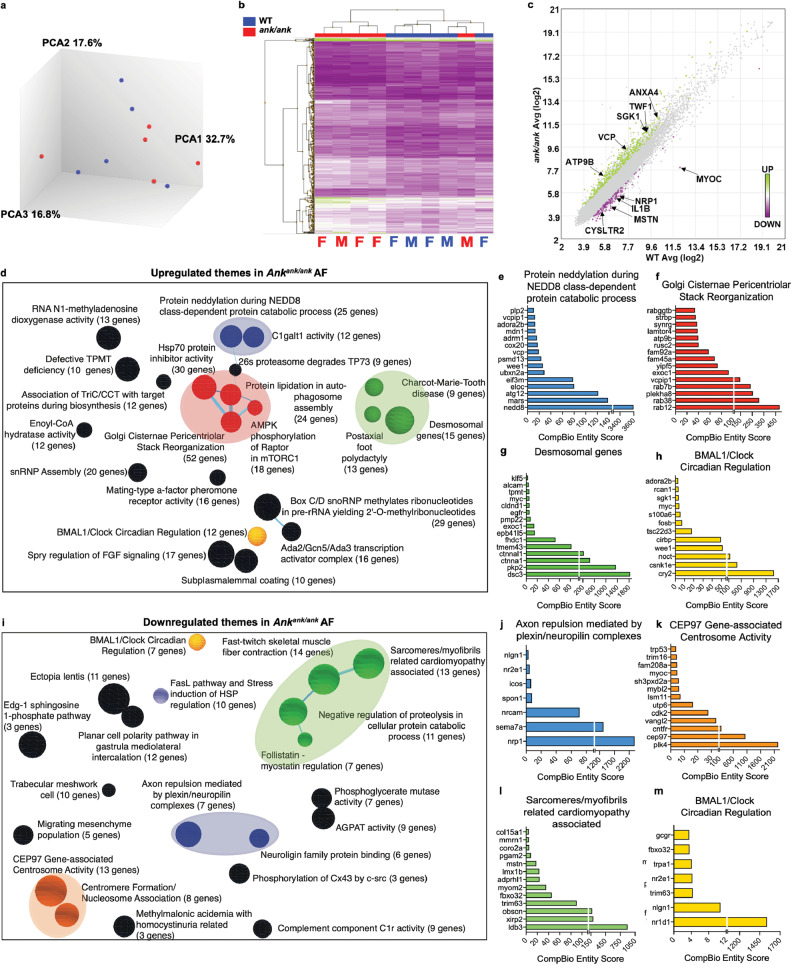
*ank* mice show dysregulated AF transcriptomic profile underscored by changes in BMAL1 circadian regulation. **a** Clustering of transcriptomic profiles by principal component analysis of the AF tissues. FFive WT mice (two male; three female) and five Mut mice (two male; three female). **b** Hierarchical clustering of significantly differentially expressed genes (DEGs) (*p* < 0.05, ≥1.75-fold change). **c** Log-log scatterplots of DEGs in the AF. **d**, **i** Themes associated with upregulated and downregulated DEGs are highlighted. The size of a sphere is related to its enrichment score and the thickness of the lines connecting themes signifies the number of genes shared between them. **e**–**h** CompBio analysis of DEGs and concepts whose abundances were significantly higher in *ank* AF cells. **j**–**m** CompBio analysis of DEGs and concepts whose abundances were significantly lower in *ank* AF cells.

We performed RNA microarray analyses of AF tissues from caudal discs of *ank* and WT mice to identify changes in gene expression induced by loss of ANK function. Three-dimensional principal component analysis (PCA) showed tight clustering based on the genotype except for one mutant sample being an outlier (Fig. 7a). In addition, gene expression profiles and the proportion of upregulated (green) and downregulated (purple) differentially expressed genes (DEGs) (*p* ≤ 0.05 and fold change ≥1.75) were discrete between WT and mutant samples by hierarchical clustering (Fig. 7b). Of 464 DEGs, 332 were upregulated and 132 were downregulated (Fig. 7c).

**Fig. 8:**
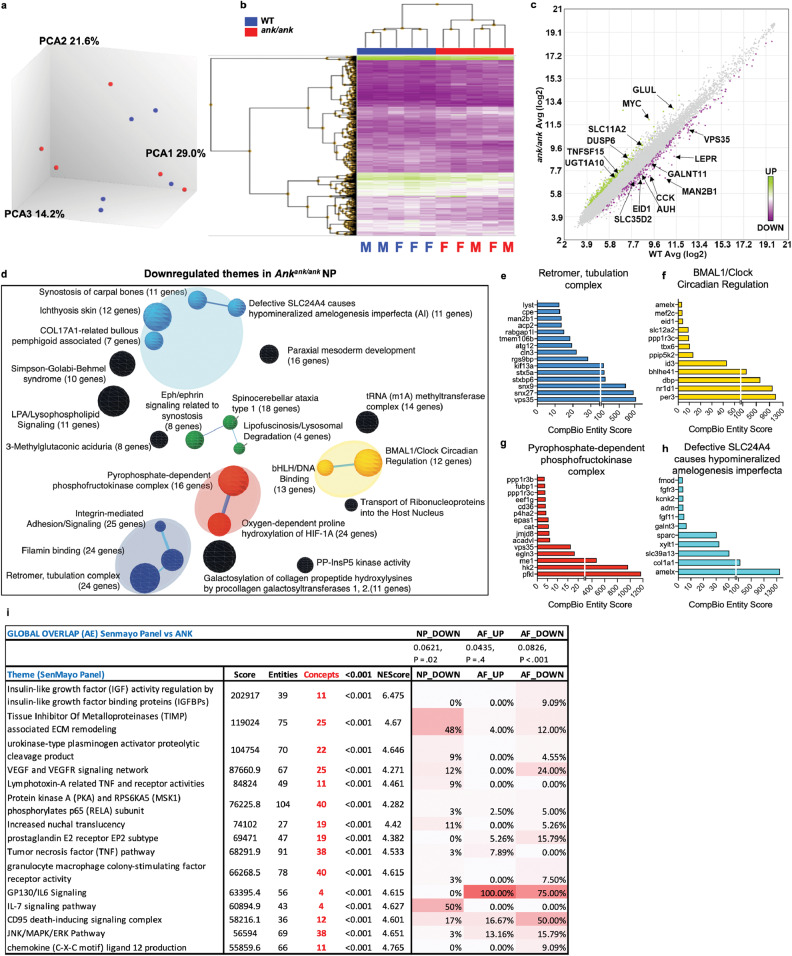
*ank* mice show dysregulation in NP transcriptome and share thematic similarities with SenMayo dataset. **a** Clustering of transcriptomic profiles by principal component analysis of the NP tissues. Five WT mice (two male; three female) and five Mut mice (two male; three female). **b** Hierarchical clustering of significantly differentially expressed genes (DEGs) (*p* < 0.05, ≥1.75-fold change). **c** Log-log scatterplots of DEGs in the NP. **d** Themes associated with downregulated DEGs are highlighted. The size of a sphere is related to its enrichment score and the thickness of the lines connecting themes signifies the number of genes shared between them. **e**–**h**. CompBio analysis of DEGs and concepts whose abundances were significantly lower in *ank* NP cells. **i** A pseudo heatmap showing global similarity, as well as theme level similarity between transcriptional profiles of *ank* NP and AF, downregulated and AF upregulated DEGs against the SenMayo gene list. Transcriptomic profiling of *ank* NP and AF downregulated themes shows commonalities with SenMayo data.

To understand the most prominent biological features associated with *Ank* loss-of-function the CompBio (PercayAI Inc., St. Louis, MO) tool was used to determine the emergent concepts and associated themes based on DEGs (Figs. 7d, i, 8d) [29]. Within the AF, the supercluster of closely related themes associated with protein modification, turnover, and catabolism *viz*. HSP70 protein inhibitor activity, protein degradation via autophagosome assembly, 26S proteasome degradation, protein neddylation, Golgi cisternae stack reorganization, and AMPK-dependent mTORC1 activity were apparent from the list of upregulated DEGs (Fig. 7d). Similarly, themes related to Charcot-Marie tooth disease, postaxial foot polydactyly, and desmosomes were present in AF upregulated genes (Fig. 7d). From the downregulated DEGs, a cluster of themes related to muscle function viz. myofibrils-related cardiomyopathy, skeletal muscle fiber contraction, negative regulation of proteolysis, and follistatin-myostatin regulation were identified (Fig. 7i). To predict the most significant genes within each theme, the top 20% (defined by thematic CompBio entity score) of contributing genes were assessed allowing for DEGs to be visualized according to their weight in the present body of literature and fold change (Figs. 7, 8 and Supplemental Figs. S4, 5). Several genes contributing to multiple themes including *nedd8*, *eloc*, *dsc3*, *pkp2*, *rab12*, *rab38*, *rab7b*, *eif4g1*, *eif3m*, *atg13*, *slc36a4, stim1*, and *stim2* were upregulated. Furthermore, AF downregulated DEGs showed enrichment into themes related to neuronal tissue: axon repulsion by plexin/neuropilin complexes, neuroligin protein family; Chromosome organization and activity: CEP97 Gene-associated centrosome activity, centromere formation/nucleosome association, and FasL pathway and stress induction of HSP regulation. These downregulated AF themes contained several enriched DEGs, including *nrp1*, *nrcam, sema7a*, *idb3*, *irp2*, *mstn*, *gdf11*, *trim63*, *fasl*, *il1b*, *fbn1*, *scarf2*, and *col15a1*.

Since NP did not evidence mineralization in young *ank* mice while showing some structural alterations, we performed microarray analyses to understand the molecular changes in the NP compartment. While three-dimensional PCA did not show a prominent distinction in the gene expression profile of WT and mutant NP samples, hierarchical clustering analysis of the proportion of upregulated (green) and downregulated (purple) DEGs (*p* ≤ 0.05 and fold change ≥1.75) showed a distinction between WT and mutant samples (Fig. 8a, b). Of the 398 DEGs, 221 genes were downregulated and 177 were upregulated between mutant vs WT NP (Fig. 8c) and used for CompBio analysis [24, 29, 30].

From the downregulated DEGs, super clusters related to (1) the ECM, pathologies related to bone and teeth mineralization and skin, (2) cell adhesion, cytoskeleton, and retromer tubulation complex, and (3) oxygen-dependent prolyl hydroxylation of HIFA signaling and PFK complex activity were identified (Fig. 8d). A select group of downregulated DEGs from the ECM super clusters included *Sparc*, *Xylt1*, *and Galnt3*. A select group of downregulated genes from oxygen-dependent proline hydroxylation of HIFA and phosphofructokinase complex/ glycolytic activity themes included *Egln3*, *P4ha2*, *Epas1*, *Jmjd8*, *Hk2*, *Pfkl*, and *Me1*. Additionally, decreased expression of genes involved in the retromer tubulation complex, such as *Vps35*, *Snx27, Snx9*, and *Kif13a*, suggested compromised endosomal transport [31]. The themes for upregulated DEGs in NP did not meet the statistical significance cutoff.

Noteworthy, both AF and NP compartments showed strong preservation of the theme “BMAL1/CLOCK circadian regulation” irrespective of the directionality of DEGs. In the AF the circadian regulation was mostly driven by *Cry2 and Nr1d1* circuit whereas, in the NP, *Per3* and *Nr1d1* contributed to this theme suggesting broader perturbation in various components of the circadian clock in *ank* mice and a plausible link to the mineralization phenotype.

### The *ank* disc transcriptomic signatures capture aspects of cell senescence

To delineate if loss of ANK function promotes cell senescence in disc tissues, we assessed the thematic overlap of DEGs from *ank* mice with SenMayo, a signature gene list that characterizes senescent cells across multiple organs including skeletal tissues (Fig. 8i) [32]. Noteworthy, the *ank* DEG signatures showed a significant global and thematic level similarity with the SenMayo dataset: AF Down *p* < 0.001; NP Down *p* < 0.02 (Supplemental Fig. S6 and Fig. 8i). We noted a thematic overlap of 48% between downregulated DEGs in NP and SenMayo dataset for themes related to TIMP-associated ECM remodeling and IL-7 signaling pathway. Similarly, AF downregulated DEGs showed a 24, 50, and 75% similarity respectively in themes associated with VEGF and VEGR signaling, CD95 death-inducing signaling complex, and gp130/IL6 signaling with the SenMayo list. Together these results suggest that loss of ANK function in the disc may affect aspects of cell senescence in both NP and AF compartments.

## Discussion

The role of ANK has been studied in skeletal tissues, which has provided insights into its biological functions in marrow stromal cell and osteoblast differentiation [13, 14, 33], acellular cementum mineralization [34–37], chondrocyte phenotype maintenance [38], and inhibition of cartilage mineralization [39]. These studies have highlighted that ANK has broader context-dependent functions than simply acting as an inhibitor of calcified mineralization. Our observation that the vertebral bone phenotype is different compared to long bones, alveolar bone, or cementum, reinforces this notion [6, 14, 35, 37].

The intervertebral disc was drastically affected by the loss of ANK function. It is likely that the difference in NP cell morphology, reduce compartment size and the increased abundance of CA3 arise from diminished load transmission through the disc due to peripheral fusion/bridge formation and generalized stress response. Further analyses of the AF revealed a higher proportion of thin fibers in the matrix, suggesting a higher collagen turnover in mutants. Decreased abundance of COL I and COMP but increased COL X in the AF highlighted the alteration of the matrix composition and decreased stability of the COL I scaffold. Together with upregulated COL X, no changes in MMP13 levels implied that differentiation of AF cells into a true hypertrophic chondrocyte-like phenotype was unlikely. A previous study suggested that COL X may inhibit calcification [23], therefore increased COL X could imply a compensatory response to inhibit dystrophic mineralization. No changes in MMP13 levels also match with the unaffected COL II abundance in the AF, since MMP13 preferentially cleaves COL II [40, 41]. There were no changes in CS and ACAN levels in the NP of mutants. However, there was a decrease in CS levels without affecting ACAN levels in the AF. This reduction in CS content in AF could be due to a decrease in other CS-rich proteoglycans such as versican or decreased CS substitution. Moreover, the existence of aligned collagen fibers in the AF is a suitable environment for mineralization, in contrast to the absence of those fibers and mineralization in the NP.

The CEP in mutants contained more TNAP-positive cells and were thicker without elevated Ki67 expression suggesting a lack of continuous cell proliferation. A previous study reported that *ank* mutants had a much larger uncalcified area in the articular cartilage of the knee joints [42]. Moreover, chondrocytes in the uncalcified area showed diffuse staining of COL X and TNAP [42], which supports our observation of thicker CEP showing TNAP-positivity. Las Heras et al. also showed that calcified cartilage in *ank* mutants was thicker and the thickness increased with age [42]. This finding was in line with our observation in the BEP showing greater BV values; it is important to point out that at 13 weeks of age, the cartilaginous template of BEP is not yet fully replaced by the bone and represents calcified cartilage.

We consider that the calcium phosphate apatite in the intermediate AF, MIA, and that of the outer AF, MOA, is different regarding their mechanism of occurrence, composition, and effect on local cells. The MIA was acellular, with high radio-opacity with peripheral cells undergoing apoptosis. Conversely, the MOA with similar X-ray density to bone contained densely residing cells, suggesting an optimal environment for cells to reside. Although imaging-FTIR spectroscopic analysis failed to show compositional similarity between vertebral bone and MOA or MIA, cells in the MOA region showed TNAP and TRAP activity. Additionally, the MOA stained positive for OCN but negative for monocyte/macrophage/osteoclast markers, suggesting the acquisition of an osteoblast-like phenotype [43]. While the lack of CD31 and IHH staining underscored that this was not an endochondral ossification process, this is the first report documenting the acquisition of osteoblast-like-phenotype by resident cells of the AF in *ank* mice.

Further insights into broader ANK function in the disc were evident from the CompBio analysis of transcriptomic signatures in *ank* mice. In the AF, ANK loss affected pathways critical in protein modifications and turnover through autophagic and 26S proteasomal pathways. Similarly, themes related to the regulation of proteolysis and stress induction of heat shock proteins were evident in downregulated DEGs implying altered protein catabolism and an overall change in cellular stress levels. Additionally, several upregulated themes in AF related to skeletal and peripheral nerve defects *viz*. foot polydactyly and Charcot-Marie-Tooth disease were indicative of the pathological relevance of these DEGs. For example, upregulated genes *stim1* and *stim2* serve as endoplasmic reticulum Ca^2+^ sensors and regulate the store-operated Ca^2+^ entry [44]. Ca^2+^ influx into terminally differentiated growth plate chondrocytes upregulates *Runx2*, *Alpl*, *Col I*, and *Ocn* expression [45], which is relevant to cellular changes seen in outer AF. Similarly, a downregulated gene *S1pr1* and its ligand S1P are reported to have an anti-inflammatory function in the disc and is downregulated during human disc degeneration suggesting an inflammation-permissive niche in *ank* mutants [46].

Sharing some similarities with AF, DEGs in NP showed enrichment in supercluster related to ECM and pathologies related to defective bone and teeth mineralization and in unique themes related to oxygen sensing through prolyl hydroxylases and cell metabolism. Dysregulation of the oxygen sensing and metabolic pathways in the disc result in functional defects, e.g., *Egln3* knockout mice showed disc degeneration and altered HIF1A signaling [47–49]. Similarly, several downregulated ECM-homeostasis-related genes including *Sparc* and *Xylt1*, are important to maintain disc health and *Sparc* knockout mice are reported to have increased incidence of herniation and endplate calcification and sclerosis [50–52]. Concerning a cluster of themes related to cell adhesion and cytoskeleton, genes such as *Flnb* have been shown to play an important role in spine health [53]. FLNB loss-of-function mutations cause multiple skeletal pathologies resulting in defects of vertebral segmentation, joint formation, and endochondral ossification [54, 55]. Further underscoring the importance of the cytoskeleton, we have shown that conditional deletion of *Arp2/Arp3*, encoding components of a major actin-cytoskeletal regulator in the cartilage and disc, results in altered cell morphology and cell fate changes [56].

One of the important features of DEGs from AF and NP tissue of *ank* mice was the prominent sharing of a thematic cluster for BMAL1/CLOCK circadian regulation, involving regulators *Cry2*, *Nr1d1*, and *Per3* suggesting that ANK loss affected different components of the circadian clock in disc cells. This is significant since disc degeneration marked by AF disorganization, increased NP vacuolation, and calcification of discs in BMAL1 knockout mice was reported underscoring the importance of this critical pathway in maintaining disc health [57, 58]. BMAL1 also regulates the mineralization of skeletal tissues including bones and teeth and negatively regulates the heterotopic ossification of tendons and ligaments with aging [59–61]. Studies have shown both an increased trabecular bone mass and mineral density in osteoblast-specific *Bmal1* knockout mice [59] and loss of bone mass affecting both cortical and trabecular bone in global *Bmal1*^*−/*−^ mice [57, 62, 63]. *Bmal*^−*/−*^ mice also show an accelerated aging phenotype and the role of BMAL1/CLOCK in cell senescence has been recognized [64–67]. Accordingly, the thematic comparison of the disc transcriptome of *ank* mice with a pronounced perturbation in BAML1/CLOCK circadian regulation with the SenMayo gene list suggested that matrix remodeling and cytokine signaling that plays a critical role in age-dependent disc degeneration were two features shared between the datasets [68–70]. Together these results suggest that loss of ANK function in the disc may affect aspects of senescence in NP and AF.

In summary, our work provides insights into disc degeneration phenotype by the loss of ANK function. It involves heterogenous carbonated calcium phosphate apatite deposition accompanied by the acquisition of osteoblast-like-phenotype in the AF, decrease in NP compartment size, and thicker CEP. The vertebral bone and bony EP were both affected but in a different fashion than other long bones and mineralized tissues (Supplemental Fig. S7). Importantly, the diversity of phenotypes in disc compartments underscores the uniqueness of this tissue compared to other cartilaginous and mineralized tissues regarding ANK function.

## Materials and methods

### Mice

All animal experiments were performed under IACUC protocols approved by Thomas Jefferson University. Mice heterozygous for the progressive ankylosis allele (*ank*) were purchased from The Jackson Laboratory (Bar Harbor, ME, USA; C3FeB6 *A/A*^*w-J*^*-Ank*^*ank/J*^, stock number 000200). Obtained Hets were used to breed *Ank*^*ank/ank*^, heterozygous and WT littermates. 4-, 13-, and 20-week-old mice were used for μCT studies whereas all remaining analyses were carried out using 13-week-old mice.

### Micro-CT analysis

Micro-CT scanning (Bruker, Billerica, MA, USA, SkyScan 1275) was performed on fixed spines using parameters: 50 kV (voltage), 200 μA (current) at 15 μm resolution. Occupation ratios of the disc mineralization were compared between WT and mutant discs. Disc height and vertebral height were measured at three different points equidistant from the center of the bone on the sagittal plane and disc height index (DHI) was calculated. The three-dimensional datasets were assessed for cortical bone analysis, and two-dimensional assessments computed cortical cross-sectional thickness (Cort. Cs. Th), cortical bone area (Cort. B. Ar), cortical bone volume (Cort. BV), and cortical bone mass density (Cort. BMD). For trabecular bone analysis, trabecular number (Tb. N.), thickness (Tb. Th), trabecular separation (Tb. Sp), trabecular BV/TV (Tb. BV/TV), and trabecular bone mass density (Tb. BMD) were computed. For bony EP analysis, EP bone volume (EP BV), EP BV/TV, EP total porosity, and EP BMD were computed.

### FTIR imaging spectroscopy

Spectral imaging data in the mid-IR region, 4000–800 cm^−1^ at 8 cm^−1^ spectral resolution and 25 μm spatial resolution was acquired from 10-μm-thick calcified T10/11 and T12/L1 disc sections from 13-week-old WT and *ank* mutant mice (*n* = 3/group) using Spotlight 400 FTIR

Imaging system (Perkin Elmer, Waltham, MA, USA). The spectral images were analyzed using ISys Chemical Imaging Analysis software (v. 5.0.0.14; Malvern Panalytical Ltd, Malvern, UK). Briefly, spectral subtraction was initially carried out to remove bands from the cryotape used for sample preparation, the spectra were smoothed (9-pt) and baseline-corrected, then single-wavenumber images reflecting the distribution of specific tissue components were acquired at 1660 cm^−1^ (protein, collagen), 960 cm^−1^ (apatite mineral, phosphate), and 870 cm^−1^ (apatite mineral, carbonate). Overlays of mineral and protein images were carried out using Fiji (ImageJ 2.1.0/1.53c) as previously described [71]. For quantification of mineral content, the mineralized regions were masked using the ISys, and the second derivative of the spectra were obtained as a mathematically objective approach to assess peak intensity [72]. The intensity ratio of the phosphate-to-amide I peak (960/1660 ratio) was calculated to inform on the relative amount of mineral-to-protein present in the tissues. The main phosphate peak at 1030 cm^−1^ was not used due to the influence of the cryotape at that spectral region. To assess the variation in mineral content across the MIA-MOA interface, the Image Topology tool on the ISys was used in regions of interest on phosphate-to-amide I second derivative peak ratio images.

### Histological analysis

Dissected spines were fixed in 4% PFA in PBS for 48 h, decalcified in 20% EDTA and embedded in paraffin. Spines used for calcified sections were fixed similarly, treated with 30% sucrose, OCT embedded, and snap-frozen. Seven-micrometer mid-coronal sections were cut from decalcified L3-S1 levels and 10 μm mid-coronal sections were cut from calcified cervical to L1/2 levels. Safranin O/Fast Green/hematoxylin staining was performed for assessing histology or Picrosirius Red staining was done to visualize the collagen content. Alizarin Red staining was used to detect calcium. Staining was visualized using an Axio Imager 2 microscope (Carl Zeiss, Jena, Germany) using 5×/0.15 N-Achroplan or 20×/0.5 EC Plan-Neofluar objectives (Carl Zeiss) or a polarizing microscope (Eclipse LV100 POL, Nikon, Tokyo, Japan) using 10×/0.25 Pol/WD 7.0 objective, Digital Sight DS-Fi2 camera, and NIS Elements Viewer software. To evaluate degeneration, mid-coronal sections from L3-S1 levels per mouse were scored using a modified Thompson Grading scale and a scoring system described by Tessier et al. [73] by at least three blinded observers [21, 74]. For Picrosirius Red staining, under polarized light, stained collagen bundles appear as either green, yellow, or red pixels that correlate to fiber thickness: green (thin), yellow (intermediate), and red (thick). Color threshold levels remained constant.

### Immunohistochemistry and analysis

De-paraffinized sections following antigen retrieval were blocked in 5–10% normal serum in PBS-T and incubated with antibodies against COL I (1:100, Millipore, Burlington, MA, USA, ABT123), COL II (1:400, Fitzgerald, North Acton, MA, USA 70R-CR008), COMP (1:200, Abcam ab231977), COL X (1:500, Abcam, Cambridge, UK, ab58632), ACAN (1:50, Millipore AB1031); CS (1:300, Abcam ab11570); CA3 (1:150, Santa Cruz Biotechnology, Dallas, TX, USA), MMP13 (1:200, Abcam ab39012), Ki67 (1:100, Abcam ab15580), OCN (1:200, Abcam ab93876), CD31 (1:400, Abcam ab124432), CD68 (1:500, Abcam ab125212), and IHH (1:120, Abcam ab39634). For CD14 (1:1000, Abcam ab182032), CTPK (1:2000, Abcam, ab37259), and ARGxx (1:200, Abcam, ab3773) staining, MOM kit (Vector laboratories, Newark, CA, USA, BMK-2202) was used for blocking and primary antibody incubation. Tissue sections were washed and incubated with Alexa Fluor-594-conjugated secondary antibody (Jackson ImmunoResearch Lab, Inc., West Grove, PA, USA, 1:500-700). The sections were mounted with ProLong® Gold or Diamond Antifade Mountant with DAPI (Fisher Scientific, Waltham, MA USA, P36934; P36966) and visualized and acquired the images with Axio Imager 2 microscope using 5×/0.15 N-Achroplan or 20×/0.5 EC Plan-Neofluar objectives (Carl Zeiss). Percent positive staining area and cell number quantification was performed using the ImageJ/FIJI software. Images were set the thresholds to subtract the background, transformed into binary, and then staining area and cell number were calculated using the analyze particles function.

### TUNEL assay

TUNEL-staining was performed using the “In situ cell death detection” Kit (Roche Diagnostic, Zug, Switzerland). Briefly, sections were de-paraffinized and permeabilized using Proteinase K (20 μg/mL) for 15 min at room temperature and a TUNEL assay was carried out per the manufacturer’s protocol. Sections were washed and mounted with ProLong^®^ Gold or Diamond Antifade Mountant with DAPI and visualized with Axio Imager 2 microscope.

### TNAP and TRAP staining

Calcified sections were prepared and stained according to the previous report [75]. Briefly, specimens attaching to the cryotape were glued on the glass slides with chitosan adhesive solution, and dried overnight at 4 °C. As for TNAP staining, slides were incubated for 10 min in 100 mM Tris-HCl buffer (AP Buffer) (pH 8–8.5) mixed with Vector Blue (Vector laboratories, SK-5300) at room temperature following the instruction of the manufacturer. Subsequently, the slides were rinsed in 1X PBS for 5 min three times and mounted with DAPI and visualized under the Cy5 channel. For TRAP staining, ELF97 (Thermo Fisher Scientific, E6588) was diluted 1:75 in TRAP buffer (9.2 g of sodium acetate anhydrous and 11.4 g of sodium tartrate dibasic dihydrate dissolved in water; total volume 1000 ml; pH 4.2). TRAP buffer was applied to the slides for 15 min at room temperature. Subsequently, ELF97 dissolved TRAP buffer was applied to the slides for minutes at room temperature. The slides were rinsed in 1X PBS for 5 min three times and mounted with DAPI and visualized under the GFP channel.

### Tissue RNA isolation

The AF and NP tissue were separately dissected from caudal discs of 13-week-old WT and *ank* mice (each *N* = 5) under a microscope (Zeiss, Stemi 503) and pooled the tissue from a single animal to serve as an individual sample. The tissue was immediately placed in RNAlater^®^ Reagent (Invitrogen, Carlsbad, CA, USA). NP and AF tissues were homogenized with a Pellet Pestle Motor (Sigma Aldrich, St. Louis, MO, USA, Z359971), and RNA was extracted from the lysates using an RNeasy^®^ Mini kit (Qiagen, Venlo, Netherlands).

### RNA microarray analysis

Total RNA with RIN of around 5 was used for the analysis. Fragmented biotin-labeled cDNA was synthesized using the GeneChip WT Plus kit (Thermo Fisher). Gene chips (Mouse Clariom S) were hybridized with biotin-labeled cDNA in 100 μL of hybridization cocktail. Arrays were washed and stained with a GeneChip hybridization wash and stain kit and scanned on an Affymetrix Gene Chip Scanner 3000 7 G, using Command Console Software. CHP files were generated by sst-rma normalization from Affymetrix CEL file using the Transcriptome Analysis Console (TAC) v4.0.2 (Affymetrix, Santa Clara, CA, USA). Only protein-coding genes were included in the analyses. The experimental group was compared to the control group in the TAC, including all probe sets where at least 50% of the samples had a DABG (detected above background) *p* < 0.05. Inclusion cutoffs were set to a 1.75-fold change and *p* value < 0.05. Analyses and visualizations were done in Affymetrix Transcriptome array console 4.0 software.

### Transcriptomic data analyses using the CompBio tool

Significantly up- and downregulated DEGs from NP and AF tissues of *ank* mice (1.75-fold, *p* < 0.05) were analyzed using the GTAC-CompBio Analysis Tool (PercayAI Inc., St. Louis, MO). CompBio performs a literature analysis to identify relevant processes and pathways represented by differentially expressed, or otherwise related, biological entities (genes, proteins, miRNAs, or metabolites). Similarly, CompBio maps were generated for the SenMayo gene set consisting of 125 genes [32]. ANK_NP_UP was a low signal dataset without any significant themes passing the *p* value threshold. So that dataset was excluded from further analysis. We performed global as well as theme-wise overlap of the SenMayo list with the three ANK datasets. For the SenMayo dataset, the top 15 themes were selected because 98.5% of input genes were mapped within those themes. Then, using the concept level overlap, similarities were calculated based on shared concepts between and amongst SenMayo and the *ank* datasets (NP Down, AF UP, and AF Down). For global similarity calculations, we used Assertion Engine (AE V1.0), a machine learning module within the CompBio tool. AE analyzes two datasets at the concept level as well as compares the interrelationships of the conserved concepts. In other words, if the same enriched concept is present in both datasets, the AE determines how similar or different that concept’s relationships are with the other concepts in the respective datasets. A global score, representing the complete contextual biological similarity between the two is computed along with a *p* value to show how far from the randomized range a given overlap is. A score of 0.0 represents no similarity and a score of 1.0 represents complete similarity [24, 29].

### ScRNA-seq data analysis

Eight-week-old rat intervertebral disc (GSE211407) [18] sample GSM6469634 and Bovine (GSE179714) [19] Sample GSM5429728 intervertebral disc single-cell RNA-seq paired-end run read one (R1) and read two (R2) FASTQ files were downloaded from European Nucleotide Archive. Using the 10x Genomics Cloud Analysis platform and single-cell 3’ gene expression library type, FASTQs were aligned to the Rattus Norvegicus or Bos Taurus references. Single-cell data analysis outputs were generated and visualized using the Loupe Cell Browser software. Rat intervertebral disc cells, endothelial cells, myeloid, lymphoid cells, and erythrocytes were identified by marker genes described [18, 19]. For human scRNA-seq, Seurat packages (version 4.3.0) was used on downloaded healthy human scRNA-seq data (GSE205535) [20].

### Statistical analysis

Statistical analysis was performed depending on the results of the D’Agostino and Pearson test and Shapiro-Wilk test to check the normality of data distribution using Prism 9.2.0 (GraphPad, La Jolla, CA, USA) with data presented as mean ± standard deviation (SD). The differences between the two groups were analyzed by *t*-test or Mann–Whitney test. The differences between the three groups were analyzed by ANOVA or Kruskal–Wallis test followed by Dunnett’s T3 multiple comparisons test or Dunn’s multiple comparison test. *p* ≤ 0.05 was considered a statistically significant difference.

## Supplementary information

Ank Supplementary Figure Legend clean

Supplementary Figure 1

Supplementary Figure 2

Supplementary Figure 3

Supplementary Figure 4

Supplementary Figure 5

Supplementary Figure 6

Supplementary Figure 7

Reproducibility Checklist

## Data Availability

RNA microarray data associated with this study are deposited in the GEO database (GSE206997). All datasets generated and analyzed during this study are included in this published article and its Supplementary Information files.
