# Supplementary material for: Loss of function mutation in Ank causes aberrant mineralization and acquisition of osteoblast-like-phenotype by the cells of the intervertebral disc
Source: Cell Death Dis. 2023 Jul 19;14(7):447. doi: 10.1038/s41419-023-05893-y (PMC10356955; doi:10.1038/s41419-023-05893-y)
Supplement: Supplementary file 1 — Ank Supplementary Figure Legend clean [file 41419_2023_5893_MOESM1_ESM.docx]

**SUPPLEMENTARY FIGURE LEGENDS**

**Supplemental Fig. S1** Representative micro-CT images of **a.** cervical and **b.** thoracic spines from wildtype (WT), *Ank* heterozygous (Het), and *ank* mutant (Mut) mice. Arrowheads indicate ectopic mineralization. Anatomical landmarks, such as the vertebra (V), transverse process (T), facet joint (F), and rib head (R) were labeled in each character. The scale bars in surface rendering images, 1mm; scale bars in multi-planer reconstruction images, 4 mm.

**Supplemental Fig. S2** Rat, bovine intervertebral discs and human nucleus pulposus tissue showing expression of *Ankh*. **a.** UMAP plot representations of rat intervertebral disc cells, endothelial cells, myeloid, lymphoid cells, and erythrocytes were identified by marker genes *Acan*, *Col1a1*, *Cdh5*, *Lyz2*, *Cd19*, and *Abcb10*. **c.** UMAP plot representations of bovine intervertebral disc cells and endothelial cells were identified by marker genes *Acan*, *Col1a1*, *Cdh5* and *Pecam1*. **b and d.** UMAP plot show *Ankh* is expressed in the disc cells. **e and f.** UMAP plot show *Ankh* is expressed in the *Acan-*expressing disc cells.

**Supplemental Fig. S3** The AF cells of *ank* do not show proliferation and do not express monocyte/macrophage/osteoclast markers nor drive endochondral ossification. **a-b.** Immunohistochemical analysis of Ki67. Scale bars, 200 μm. N = 1-4 discs/mouse, 6-9

mice/genotype. Dotted lines demarcate different tissue compartments within the disc. Quantitative measurements represent mean ± SD. Significance was tested using Kruskal-Wallis test. Qualitative immunohistochemical analyses of **c.** cathepsin K (CTSK); **d.** CD68; **e.** CD14; **f.** CD31; **g.** Indian hedgehog (IHH). **c-e.** Scale bars, 200 μm for the discs and 25 μm for the positive controls; **f.** All the scale bars, 25 μm. **g.** Scale bars, 100 μm for the discs and 25 μm for the positive controls. Dotted lines demarcate different tissue compartments within the disc. Staining of positive controls are shown from indicated regions.

**Supplemental Fig. S4** The upregulated and downregulated themes and corresponding DEGs with their CompBio entity scores in *ank* AF cells. **a-g.** CompBio analysis of DEGs and concepts whose abundances were significantly higher in *ank* AF cells. **h-n.** CompBio analysis of DEGs and concepts whose abundances were significantly lower in *ank* AF cells.

**Supplemental Fig. S5** The downregulated themes and corresponding DEGs with their CompBio entity scores in *ank* NP cells. **a-f.** CompBio analysis of DEGs and concepts whose abundances were significantly lower in *ank* NP cells.

**Supplemental Fig. S6** The global similarity scores for *ank* and SenMayo using the assertion engine tool within CompBio. **a.** *ank* NP and SenMayo downregulated **b.** *ank* AF and SenMayo downregulated; **c.** *ank* AF and SenMayo upregulated. The background distribution (blue dots) is generated by comparing real datasets of various sizes with random gene lists. The orange points show the scores generated by datasets of sizes similar to the input datasets used for p-value calculations. The red dot (highlighted) shows the global similarity between the *ank* and SenMayo cluster comparison.

**Supplemental Fig. S7** A scheme of pathological condition of the intervertebral disc and spine induced by loss of *Ank* function.
