# Supplementary figures and images for: Loss of function mutation in Ank causes aberrant mineralization and acquisition of osteoblast-like-phenotype by the cells of the intervertebral disc
Source: Cell Death Dis. 2023 Jul 19;14(7):447. doi: 10.1038/s41419-023-05893-y (PMC10356955; doi:10.1038/s41419-023-05893-y)

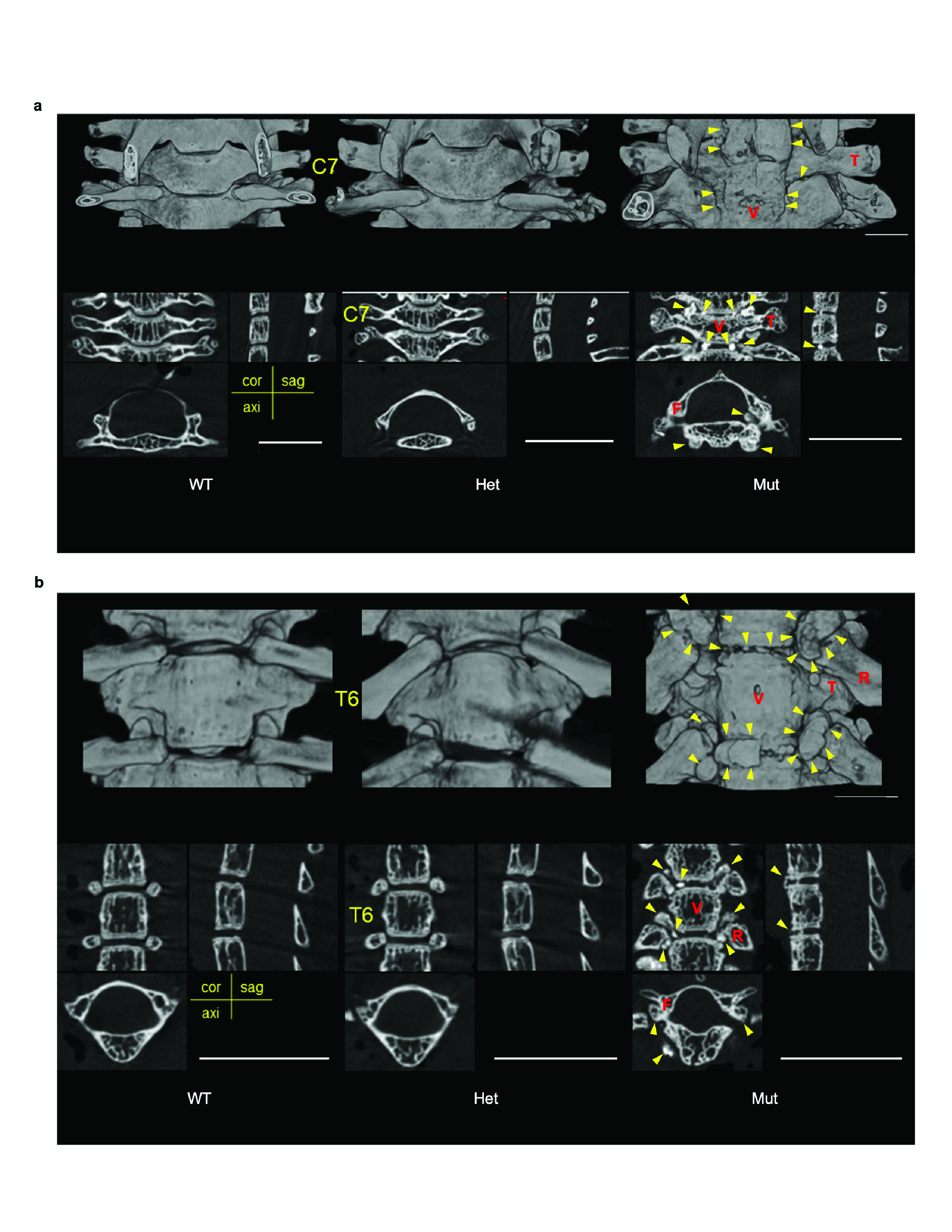

Supplement: Supplementary file 2 — Supplementary Figure 1 [file 41419_2023_5893_MOESM2_ESM.tif]

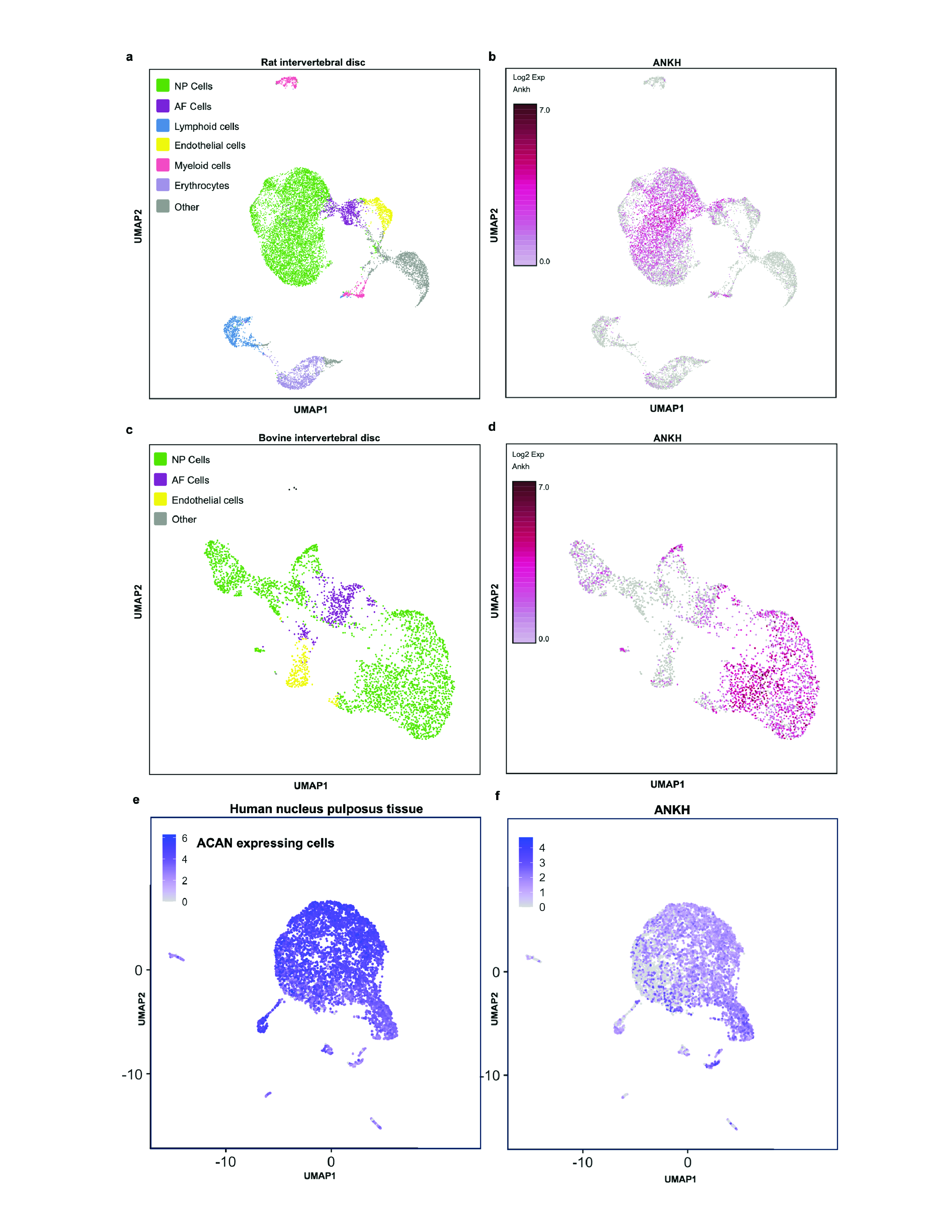

Supplement: Supplementary file 3 — Supplementary Figure 2 [file 41419_2023_5893_MOESM3_ESM.tif]

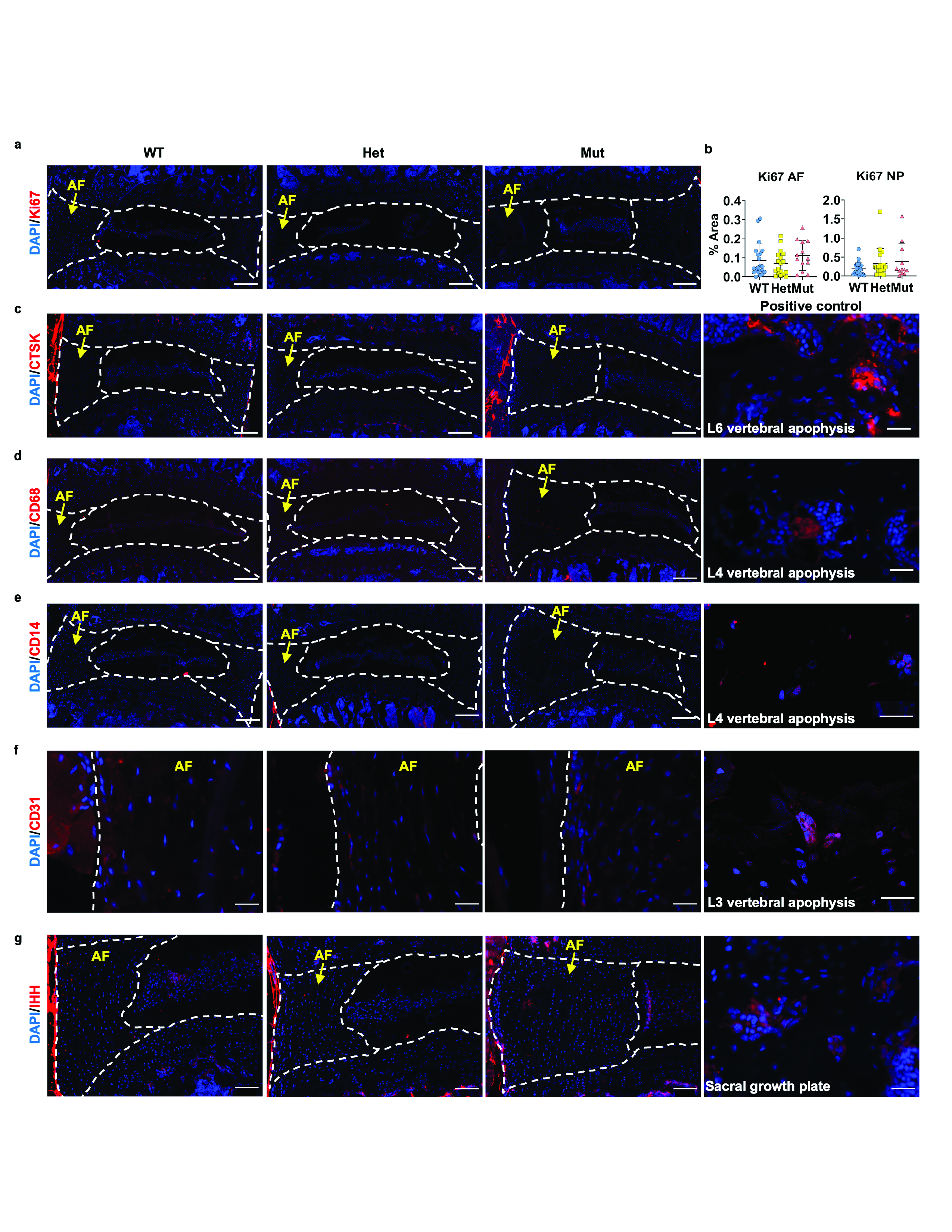

Supplement: Supplementary file 4 — Supplementary Figure 3 [file 41419_2023_5893_MOESM4_ESM.tif]

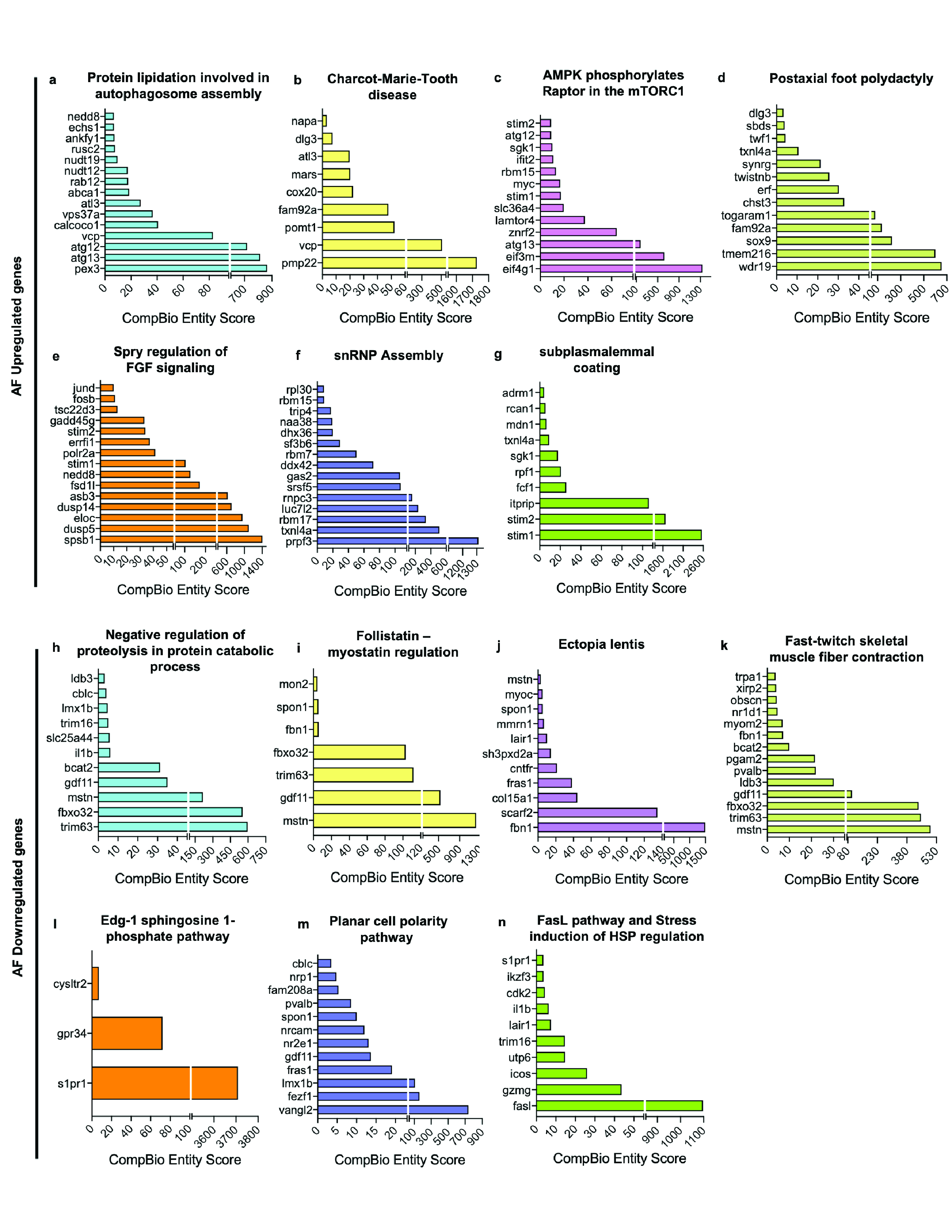

Supplement: Supplementary file 5 — Supplementary Figure 4 [file 41419_2023_5893_MOESM5_ESM.tif]

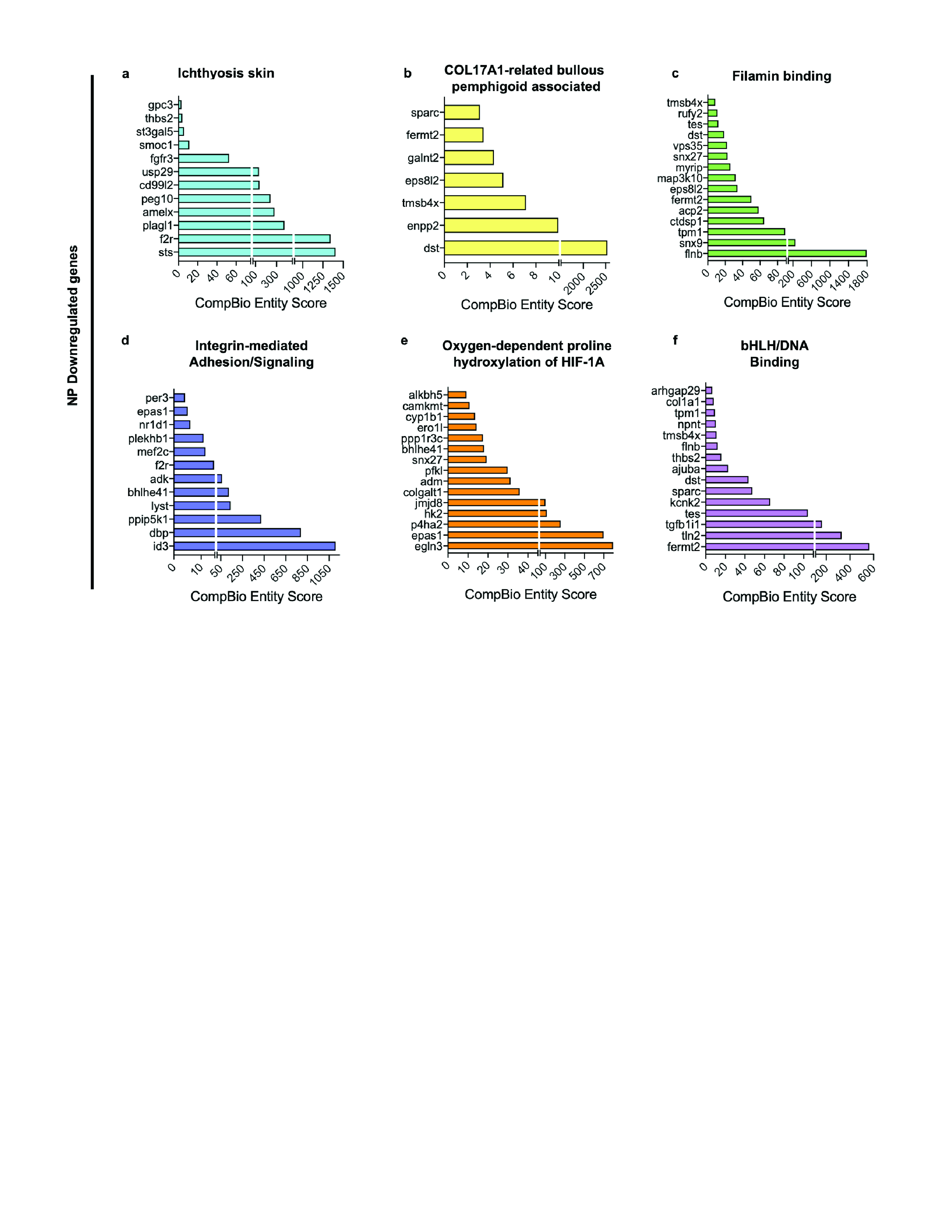

Supplement: Supplementary file 6 — Supplementary Figure 5 [file 41419_2023_5893_MOESM6_ESM.tif]

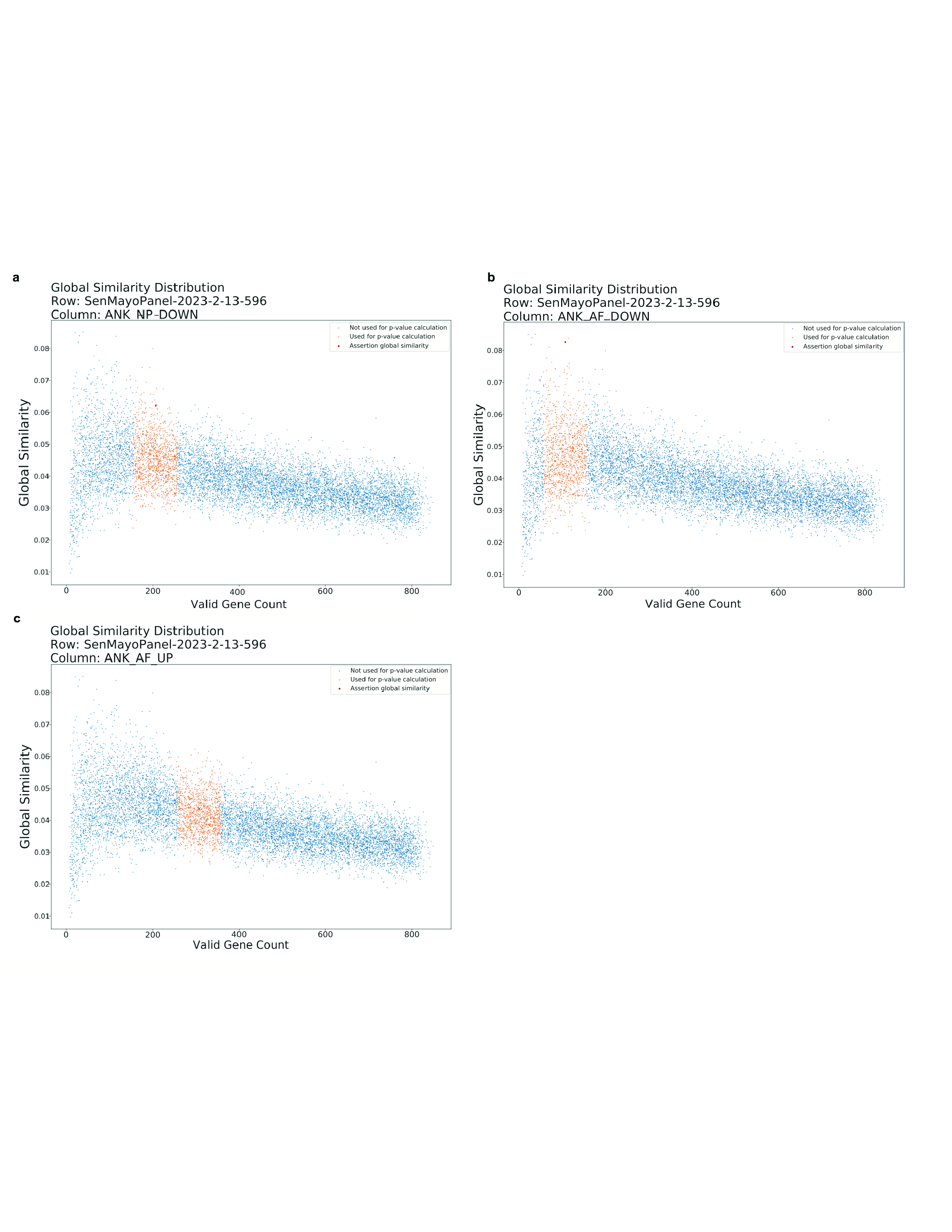

Supplement: Supplementary file 7 — Supplementary Figure 6 [file 41419_2023_5893_MOESM7_ESM.tif]

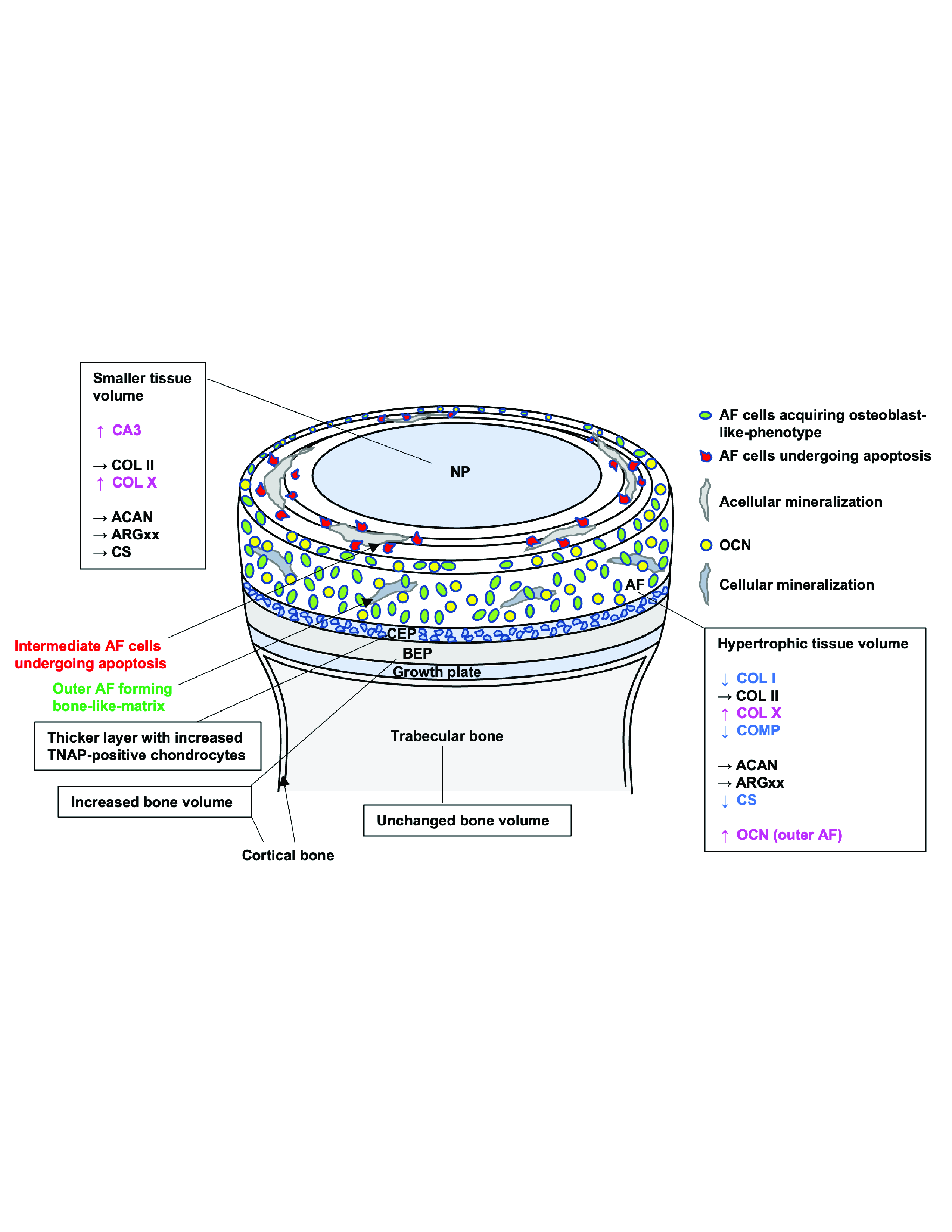

Supplement: Supplementary file 8 — Supplementary Figure 7 [file 41419_2023_5893_MOESM8_ESM.tif]
